# Mandibular morphometric analysis in open bite early treatment relapse subjects: a retrospective observational pilot study

**DOI:** 10.1186/s12903-022-02546-y

**Published:** 2022-12-01

**Authors:** Valeria Paoloni, Letizia Lugli, Carlotta Danesi, Paola Cozza

**Affiliations:** 1 Department of Systems Medicine, University of Rome Tor Vergata, Viale Oxforx, 81, 00133 Rome, Italy; 2 Department of Dentistry UNSBC, Tirana, Albania; 3 UniCamillus-Saint Camillus International University of Health Sciences, Rome, Italy

**Keywords:** Anterior open bite, Early orthodontic treatment, Treatment relapse features, Mandibular shape analysis

## Abstract

**Background:**

The purpose of this retrospective study was to evaluate the mandibular shape differences between a group of success and a group of failure Anterior Open Bite (AOB) malocclusion early orthodontic treatment in growing subjects, in order to identify mandibular features of relapse.

**Methods:**

Twenty three patients (7 males, 16 females, 9.3 years ±1,5 years) were enrolled from the Department of Orthodontics at the University of Rome Tor Vergata. Inclusion criteria were: white ancestry, overbite < 0 mm, mixed dentition phase, end-to-end or Class I molar relationship, first skeletal class assessed on lateral cephalograms (0° < ANB < 4°), cervical skeletal maturation CS1-CS2, no previous orthodontic treatment, no congenital diseases. Pre-treatment (T1) lateral cephalograms were acquired. Each patient underwent early orthodontic treatment with Rapid Maxillary Expander (RME) and Bite Block (BB) or Quad-Helix Crib (QHC) until open bite correction. Radiographic records were recollected at T2 (permanent dentition, skeletal cervical maturation CS3-CS4). Mean interval time T2-T1 was 4.2 years ±6 months. According to treatment stability, a Relapse Group (RG 11 patients, 3 M, 8F; 13.7 years ±8 months, 7 subjects treated with RME/BB, 4 with QH/C) and a Success Group (SG, 12 patients, 4 M, 8F; 13.4 ± 10 months, 7 subjects treated with QH/C, 5 with RME/BB) were identified. On the lateral radiographs the mandibular length (Co-Gn), the inferior gonial angle (NGo^GoMe) and the antegonial notch depth (AND) were analyzed. Then the mandibular Geometric Morphometric analysis (GMM) was applied. Intergroup statistically significant differences were found using student’s *t*-tests. Procrustes analysis and principal component analysis (PCA) were performed for the GMM.

**Results:**

At T1 no statistically significant differences were found between RG and SG, however higher values of antegonial notch depth were found in RG. T2-T1 comparison showed in RG statistically significant increases in Co-Gn (*p* = 0.04), NGo^GoMe angle (*p* = 0.01) and antegonial notch depth (*p* = 0,04). PC1 confirmed the increase in the antegonial notch depth in RG when compared to SG at T2.

**Conclusions:**

The increased antegonial notch depth associated with the increased mandibular length and the increased gonial angle could be responsible of relapse of early orthodontic treatment in open bite growing subjects.

**Supplementary Information:**

The online version contains supplementary material available at 10.1186/s12903-022-02546-y.

## Introduction

The anterior open bite (AOB) is defined as an alteration in the vertical relationship between the maxillary and mandibular dental arches, characterized by a negative overbite that is a lack of contact between the upper and lower incisal edges in occlusion [1–3].

The main goal of the orthodontic therapy in these cases is to achieve a long-term stability of occlusion especially on the vertical plane. Indeed, among the various types of malocclusions, AOB is historically considered one of the most challenging to treat and its correction is prone to relapse [4].

The principal opponent to long-term treatment stability is the unfavourable residual growth potential acting after the end of treatment. Moreover, counted with the craniofacial skeletal characteristics of AOB patients, a backward mandibular rotation and a vertical mandibular growth pattern are usually associated with open-bite relapse [5, 6].

According to the different aetiology of the malocclusion, a broad diversity in terms of therapeutic approaches has been proposed in the early management of AOB. These treatment modalities include functional appliances as open bite Bionator, Frankel appliance or Teuscher appliance, or multibracket techniques, headgears, fixed or removable palatal crib and bite blocks [7, 8]. Among them, Quad-helix/crib appliance (QH/C) is used to treat dentoskeletal open bite usually associated to non-nutritive sucking habits [9], while skeletal AOB should be managed early in growing subjects by applying Rapid Maxillary Expansion (RME) in association with a posterior bite block (BB) to control the vertical dimension by avoiding the extrusion of both lower and upper molars [10–12]. As reported in literature, both these early therapeutic protocols led to successful and long-term stable recovery of positive overbite [13, 14]. It can be assessed that fixed palatal cribs showed a greater amount of overbite improvement compared to removable appliances [8].

Despite the early interceptive therapy carries a greater possibility of resolving these skeletal, dental and functional imbalances in AOB, the risk of relapse is still present and the vertical growth pattern with a downward rotation of the mandible is usually correlated with open bite treatment failure.

In literature different studies about relapse in AOB are present [1–6, 15, 16] However, to our knowledge no data are available about the morphological variation of the mandible between AOB early treatment success and relapse patients.

This study is based on the hypothesis that the open bite relapse is caused by a morphological change of the mandible resultant to residual growth potential acting after the end of the treatment.

A method of visualization of shape changes proposed in literature is the Geometric Morphometric analysis (GMM) [17, 18]. Previous studies used GMM to assess the morphological shape variations of the palatal vault in AOB growing subjects when compared with a control group or to evaluate the morphological mandibular and palatal characteristics in AOB treated subjects compared with an untreated control group at the end of growth [19–21].

Aim of this retrospective study was to analyse the morphological mandibular characteristics after early interceptive treatment in AOB subjects by comparing a successful group to a failure one through the means of conventional cephalometric analysis and geometric morphometric analysis in order to evaluate mandibular features of relapse.

## Materials and methods

### Study design, setting, participants

This project was approved by the Ethical Committee of the University of Rome Tor Vergata (Protocol number: 248/20) and parents of all subjects included in the study signed the informed consent.

Among 330 patients with AOB malocclusion who received early orthodontic treatment from January 2010 to December 2016, a sample of 23 patients (AOB group, 7 males, 16 females, 9.3 years ±1.5 years) was retrospectively enrolled from the Department of Orthodontics at the University of Rome Tor Vergata.

Subjects included in the study group were selected according to the following inclusion criteria: European ancestry (white), anterior open bite (− 4 < OVB < 0 mm, extending from incisors to canine), increased vertical dimension as assessed on lateral cephalograms (SN^GoGN > 37°), mixed dentition phase with fully erupted first permanent upper molars, end-to-end or Class I molar relationship, first skeletal class assessed on lateral cephalograms (0° < ANB < 4°), prepubertal skeletal maturation (CS1-CS2) [22], good quality of pre-treatment and post-treatment study casts, follow up until they reached skeletal cervical maturation CS3-CS4 with all their permanent teeth erupted except for the third molars.

Exclusion criteria were: previous orthodontic treatment, appliance breakage, multiple and/or advanced caries, tooth agenesis, supernumerary teeth, cleft lip and/or palate and other genetic diseases, no follow up.

At pre-treatment phase (T1), radiographic records were acquired (panoramic and lateral cephalograms) in order to make a right diagnosis and decide the correct treatment plan.

The AOB group received two types of early interceptive treatment according to the presence or absence of prolonged sucking habits. When the sucking habit was not recorded and there was a skeletal AOB, the patient was treated by RME/BB (RME/BB group, 12 subjects), otherwise when the sucking habit was observed the subject was treated by QH/C (QH/C group, 11 subjects) [19].

**Fig. 1:**
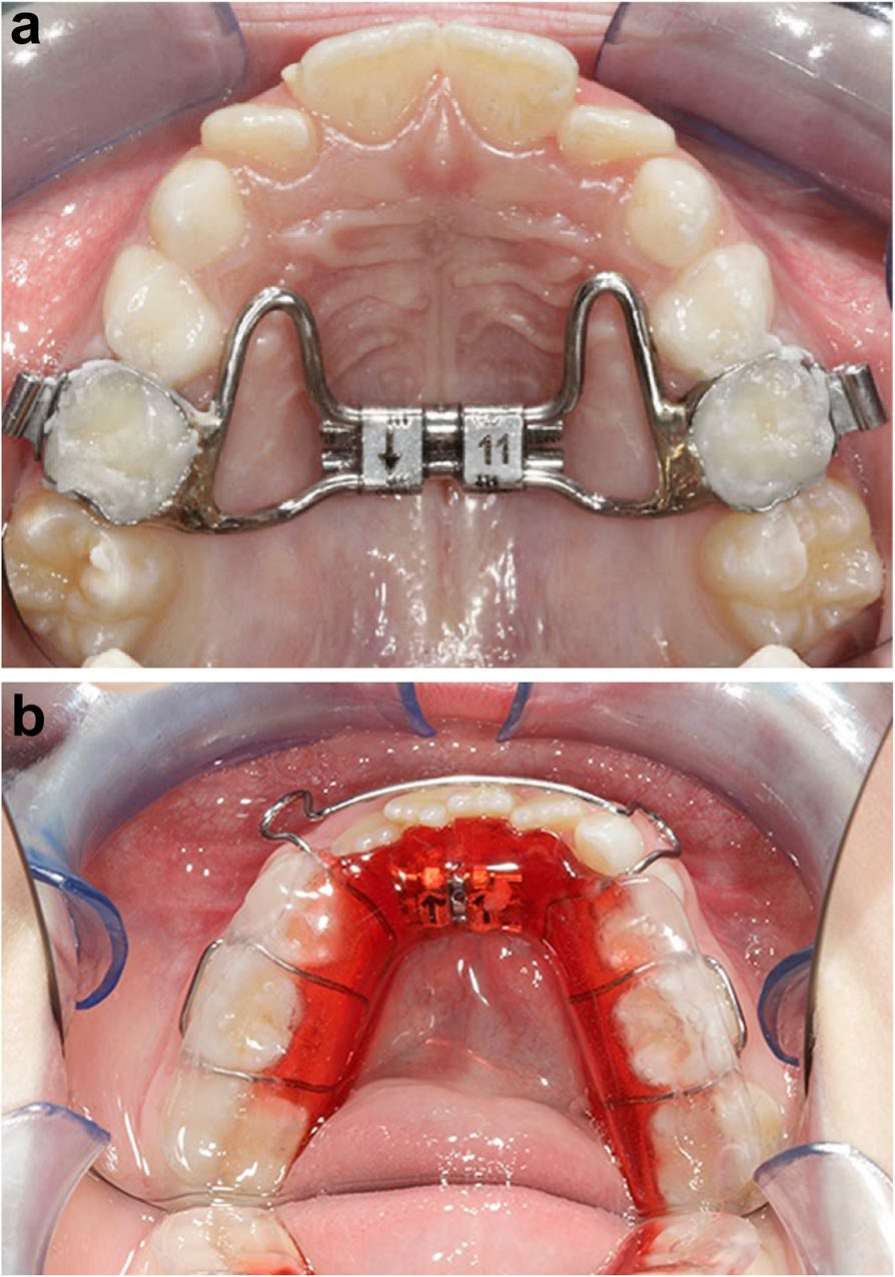
**a**, **b** Rapid Maxillary Expander and Bite Block treatment

Each subject of the RME/BB group underwent a therapy with RME soldered to bands on the first permanent molars or on the second deciduous molars. The expansion screw (Leone SpA; Sesto Fiorentino, Florence, Italy) was turned one time a day until the palatal cusps of the upper posterior teeth approximated the buccal cusps of the lower posterior teeth; then the appliance was left in place for at least 8 months as a passive retainer to make stable the expansion reached during screw activation. After RME removal, no other device was prescribed to the patient. The BB appliance was projected in the form of a Schwartz device for the mandibular arch with resin splints of 5-mm thickness in the posterior occlusal region. The BB was applied for 12 months to control the vertical dimension. The patients wear the BB 20 hours a day (Fig. 1a-b). Their compliance was assessed with a face-to-face interview conducted by a single investigator by using a 3-point Likert-type scale (poor, moderate, and good) [23] at the end of the treatment: poor compliance was declared when the patient wore the BB at night only, moderate compliance happened when the patient wore the BB at night and during the day at home, and good compliance was established when the patient wore the BB full time as suggested by the clinician [19].

**Fig. 2:**
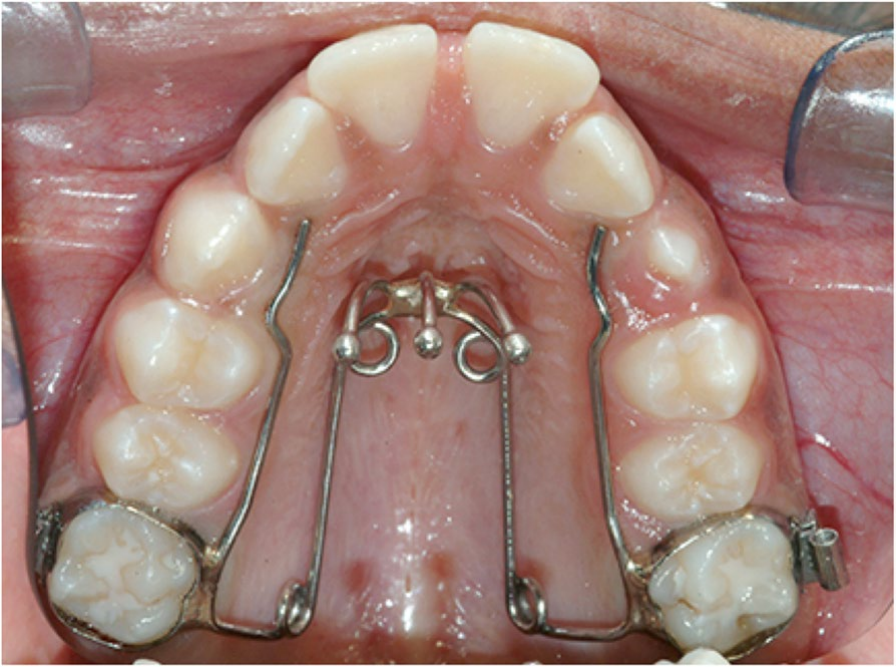
Quad Helix with Crib treatment

Each subject of the QH/C group underwent an early therapy with a QH/C made of 0.036-in stainless wire soldered to bands on the first permanent molars or on the second deciduous molars. QH/C activation was correspondent to the buccolingual width of 1 M. The device was reactivated once or twice during therapy to reach overcorrection of the transverse relationships (Fig. 2).

All the subjects were treated by two operators with more than 10 years of experience.

At the end of this first phase of treatment (mean treatment time 15 months ±3 months), all the patients reached the open bite malocclusion correction with positive overbite, eventually oral habits/dysfunction were removed, and they underwent routine recalls to follow-up treatment stability. These patients were seen every 6 months until they reached a skeletal cervical maturation CS3-CS4 with all their permanent teeth erupted except for the third molars (T2), in mean 4.2 years ±6 months after T1.

According to the treatment stability of early treatment, two groups were clinically identified: Relapse Group (RG) and Success Group (SG). RG (11 patients, 3 M, 8F; mean age: 13.7 years ±8 months), 7 subjects treated with RME/BB, 4 with QH/C) presented at T2 an anterior open bite malocclusion and needed a new orthodontic treatment; SG (12 patients, 4 M, 8F; mean age: 13.4 ± 10 months), 7 subjects treated with QH/C, 5 with RME/BB) didn’t present an anterior open bite malocclusion but good occlusal parameters.

### Measurements variables

For each patient headfilms were collected at T1 and T2 using a modern cephalostat with 1.5 m of focus/film distance.

All the cephalograms were standardized with regard to magnification factor by setting this at 0%. Cephalometric software (Viewbox, version 4.0, dHAL Software, Kifissia, Greece) was used for the lateral radiographs evaluation.

**Fig. 3:**
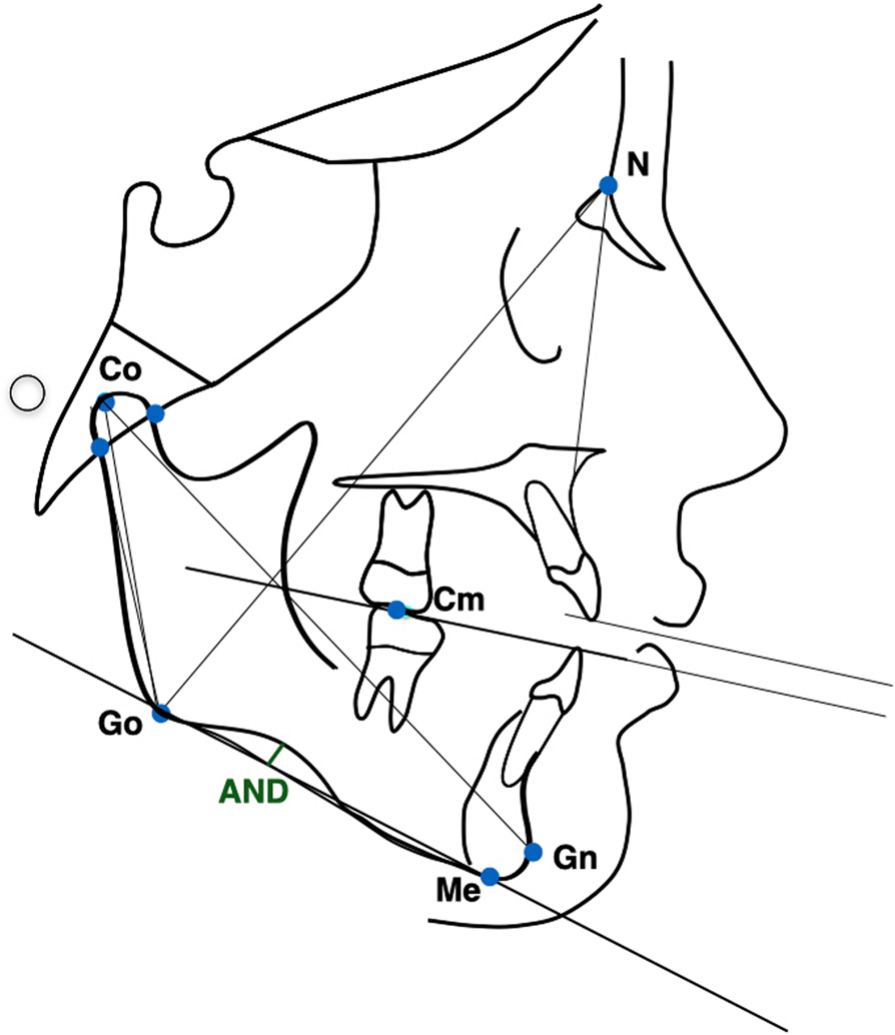
Cephalometric points, lines, and angles used in analysis

At T1 cephalometric analyses were conducted to assess the overlap of the two groups RG and SG. The cephalometric reference points, lines and angles used in the analysis are shown in Fig. 3. The analysed measurements were the mandibular length (Co-Gn), the inferior gonial angle (NGo^GoMe) and the antegonial notch depth (AND). The antegonial notch depth was evaluated by drawing a line between the anterior convexity point (ACP: the point of greatest convexity along the anterior-inferior border of the mandible) and the inferior gonion (IGo: the point of greatest convexity along the posterior-inferior border of the mandible). Antegonial notch depth is the distance between the greatest point of convexity in antegonial notch area in the lower border of mandible and the line described above.

**Fig. 4:**
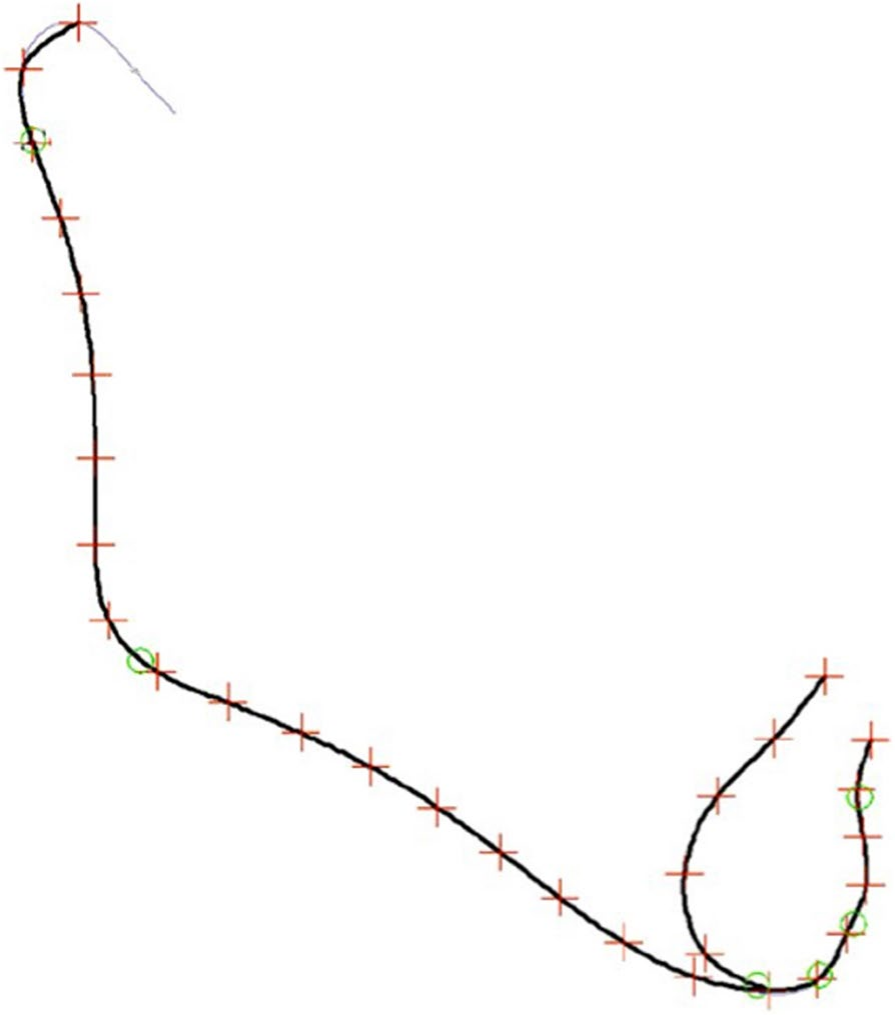
Fixed landmarks (green circles) and sliding semilandmarks (red crosses) used to describe the mandible

To study mandibular shape, GMM was applied using Viewbox software (version 4.0, dHAL Software, Kifissia, Greece). For the evaluation of the shape of the mandible 2 continuous curves with 31 points, 6 of them being fixed cephalometric landmarks, were drawn (Fig. 4). The remaining landmarks were semilandmarks, initially placed at equidistant distances along the curves. The averages of all the mandibular datasets were calculated and used as a fixed reference (Procrustes average) to allow all semilandmarks to slide and become more homologous from subject to subject in order to minimize the thin-plate spline bending energy [24, 25]. This procedure was redone twice.

### Statistical analysis

In a pilot study 10 patients were used to calculate the reproducibility and the sample size which indicated the need for approximately 22 patients to estimate the mandibular length (Co-Gn) with a 95% confidence interval (CI); a minimum difference of 2.5 mm and a standard deviation (SD) of 2.5 mm, with a power of 80%.

To determinate the method accuracy, one trained examiner (LL) with an experience of 4 years performed all the measurements on lateral cephalograms and 20 radiographs were retraced after an interval of approximately 2 weeks. A paired t-test was used to compare the two measurements (systematic error, *p*-value < 0,05). Sample normality was tested using the Shapiro-Wilk test.

In the presence of normally distributed data, descriptive statistics were calculated for each measurement in each group and significant between-group differences were tested with the independent sample Student’s *t*-test at T1 and at T2.

The primary outcome of the study was the difference in mandibular length (Co-Gn) measured at baseline (T1) and after the follow-up (T2) on the lateral cephalograms. Secondary outcomes were the T2-T1 differences in the variables measured on lateral cephalograms.

For the GMM analysis, Procrustes analysis was applied and principal component analysis was performed to reveal the main patterns of mandibular shape variation. Procrustes distance between group means was used to evaluate the statistical differences between the groups at T2. More than 10,000 permutations have been reported [19].

## Results

No systematic error was found between the repeated cephalometric values; while the mean random error of the 20 repeated digitizations for the geometric morphometric analysis, expressed as a percentage of total shape variance, was 3.7%.

Mean interval time of observation T2-T1 was 4.2 years ±6 months.

At T1 all the patients presented anterior open bite (mean OVB value: − 4.41 mm), while at T2 only the RG subjects had anterior open bite (mean OVB value: − 2.23 mm).

No significant gender difference between groups was found.

Comparison of pre-treatment (T1) values between RG and SG showed none statistically significant differences (Table 1).

**Table 1:** Descriptive Statistics and Statistical Comparisons (unpaired Student’s t-tests) of the intergroup differences between Relapse Group (RG) and Success Group (SG) at T1 (*p*-value < 0.05)

Variables	Relapse Group (RG) (***n*** = 11)		Success Group (SG) (***n*** = 12)		Diff (RG-SG)	95% CI	***P*** value
	Mean	SD	Mean	SD			
Co-Gn	96.89	2.97	95.61	2.85	1.28	−1.2442 to 3.8042	NS 0.302
NGo^GoMe	81.37	3.43	79.92	4.9	1.45	−2.2512 to 5.1512	NS 0.418
Antegonial Notch Depth	2.07	0.65	1.51	0.66	0.56	−0.0088 to 1.1288	NS 0.053

At T2 RG, when compared to SG, showed statistically significant increases in the mandibular length Co-Gn (*p* = 0.045), in the inferior gonial angle NGo^GoMe angle (*p* = 0.0345) and in antegonial notch depth (*p* = 0.022) (Table 2).

**Table 2:** Descriptive Statistics and Statistical Comparisons (unpaired Student’s t-tests) of the intergroup differences between Relapse Group (RG) and Success Group (SG) at T2 (*p*-value < 0.05)

Variables	Relapse Group (RG) (***n*** = 11)		Success Group (SG) (***n*** = 12)		Diff (RG-SG)	95% CI	***P*** value
	Mean	SD	Mean	SD			
Co-Gn	99.97	3.11	97.18	3.15	2.79	0.0720 to 5.5080	*0.045
NGo^GoMe	83.76	3.86	80.49	3.09	3.27	0.2508 to 6.2892	* 0.035
Antegonial Notch Depth	2.45	0.69	1.58	0.96	0.87	0.1388 to 1.6012	* 0.022

**Fig. 5:**
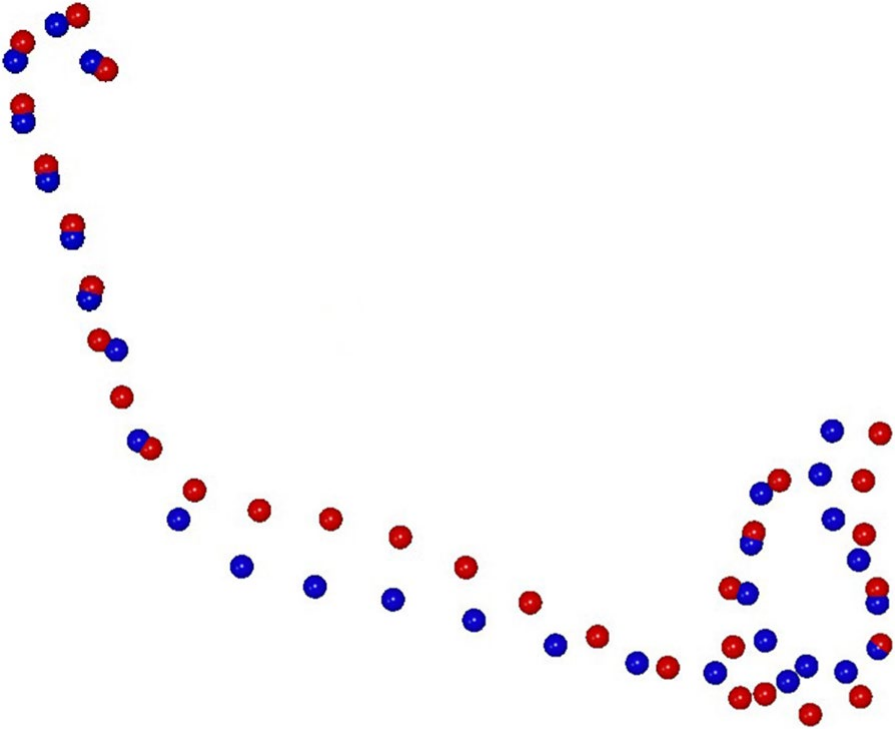
Morphological mandibular comparison between RG (red) and SG (blu) at T2

For the changes in the mandibular morphology, a statistically significant difference between RG and SG mandibular shape was found at T2 (10,000 permutations; *P* = 0.046) (Fig. 5). The first principal component (PC1) explained the largest variance and was morphologically considered to be the most meaningful.

By comparing RG and SG groups, the variation described by PC1 defined the 47% of total shape variance. PC1 showed significant changes in the antegonial notch area with an increased in depth in RG compared to SG.

## Discussion

Early treatment of AOB malocclusion is able to intercept and reduce the dentoskeletal open bite [8]. However, the risk of relapse is still present and dentoskeletal posttreatment changes seem to continue toward open-bite relapse [26, 27]. In fact, the vertical growth pattern with a downward rotation of the mandible is usually correlated with open bite treatment failure.

The few studies that focus on this topic primarily evaluate success rate treatment without studying predictors of relapse. Remmers et al. [28] observed that 27% of 52 successfully treated patients showed opening of the bite 5 years after treatment; Jonson et al. [29] showed negative overlap in 25.8% of their sample group at the end of post-treatment period, Lopez Gavito et al. [6] found treatment relapse in more than 35% of their patients in the post-retention period, while Hang et al. [30] in 17.4% of no growing analysed subjects. Most of them investigate the post-retention stability of fixed appliance treatment or of orthognathic surgery in non-growing subjects, but they didn’t analyse early interceptive treatment of the malocclusion [31].

Their outcomes reported that the only one common relapse factor is the vertical growth pattern with a downward rotation of the mandible. These failure patients exhibited a mean mandibular plane and gonial angles higher and a mean posterior facial height ratios lower than the success ones. Moreover, they showed an increase in posterior maxillary facial height, resulting in downward rotation of the mandible [29].

As previously underlined, no study succeeded in finding predictors of AOB treatment relapse and this leads to the overall conclusion that open bite cannot be successfully predicted from the pre-treatment cephalometric variables. As stated by Remmers et al. [28], long-term stability of the open bite correction is not a matter of treatment method or appliance, but it is mainly influenced by growth after treatment or by functional disturbances. In the individuals who have an excessive growth of the lower anterior face height the mandible shows backward rotation, with an increase in the mandibular plane angle. This type of rotation is associated with anterior open bite malocclusion and mandibular deficiency (because the chin rotates back as well as down) [29].

The purpose of the present study was to evaluate the mandibular shape modifications between a success group (SG) and a failure group (RG) of anterior open bite malocclusion early orthodontic treatment in growing subjects, in order to identify mandibular features of relapse. In fact, shape changes could make different responses to orthodontic therapy.

In literature different approaches to the correction of AOB are reported: functional appliances as open bite Bionator, Frankel appliance or Teuscher appliance, or multibracket techniques, headgears, fixed or removable palatal crib and bite blocks. Showkatbakhsh et al. [32] proposed a combined approach to treat a complex case of a 12-year-old boy with an open bite treated with a hyrax combined with fixed tongue appliance mounted in the upper jaw, a posterior bite plate mounted in the lower jaw and a reverse chin cup.

In our study the two orthodontic treatments proposed were the Quad-helix/crib appliance (QH/C) and the Rapid Maxillary Expansion with posterior bite block (BB). QH/C is used when there is a non-nutritive sucking habits in order to solve dentoskeletal open bite [9], while RME/BB managed skeletal AOB in early growing subjects by applying expansion in association with a posterior control of the vertical dimension by avoiding the extrusion of both lower and upper molars [10–12].

Our results show that before early orthodontic treatment, RG and SG were comparable for all the examined variables.

At T2, RG presents significant increases in the mandibular length (*p* = 0.045), inferior gonial angle (*p* = 0.035) and antegonial notch depth (*p* = 0.022). These mandibular shape differences are also highlighted by the means of GMM that visually shows the deeper antegonial notch of the RG (*p* = 0.046).

### Significant changes of the mandibular length

(Co-Go and Co-Gn) were also observed in other studies. Janson et al. [5] explained the increase of the mandibular length as the presence of a remaining intrinsic mandibular growth during the postretention period after fixed appliance treatment of AOB growing subjects that represents a relapse factor. Since the aim of the AOB therapy is to correct the malocclusion on the vertical plane, changes on the sagittal plane of the mandible are not expected and it is very unlikely that these modifications are related to significant decrease of the anterior overbite. These findings allowed an association between relapse features of open bite malocclusion to relapse features of Class III malocclusion. In fact, unsuccessful Class III early treatment subjects usually presented an excessive growth of the facial height, a hyperdivergent pattern, larger gonial angle and increase in the total mandibular length.

### The second finding of our study was the increased inferior gonial angle

The obtuse gonial angle was associated with a skeletal open bite relapse due to an increase in posterior maxillary facial height that causes a higher posterior dentoalveolar maxillary height and a forerun posterior dental contact with a significant bite-opening downward and backward rotation of the mandible [33, 34]. This modification of the posterior occlusal dental contacts remodels the posterior part of the mandibular body acting not only on the gonial region of the mandible but also on the antegionial region.

### Therefore, the increased inferior gonial angle is related to the increased antegonial notch depth

The antegonial notching has been an interesting topic for studying growth and development of the mandible and it has been associated with different facial characteristics. Mandibular antegonial notch is present on the lower margin of the mandibular body, at the junction between the ramus and the body of the mandible, immediately anterior to its angle. It has been observed in Bjork’s study that apposition beneath the gonial angle together with excessive resorption under the symphysis in mandibles with backward and downward rotation results in upward curving of the inferior border of the mandible anterior to the angular process (gonion) and is known as antegonial notching. The antegonial notch region is an indicator of mandibular growth potential and its depth is usually associated to backward pattern of mandibular rotation and a vertical direction of mandibular growth [33]. Therefore changes in its morphology are important to evaluate treatment stability. Deep antegonial notching leads to a backward pattern of mandibular rotation and a vertical direction of mandibular growth. In GMM, PC1 showed significant mandibular shape changes with an increased inferior gonial angle and an increased antegonial notch depth of the RG at T2. However, our analysis does not imply that our model is able to classify good responder or bad responder, but it could help to guide clinicians to intercept long-term mandibular features of AOB relapse. The analysis and the visualization of the mandibular morphology, especially of the antegonial notch depth, on the lateral radiographs during the orthodontic treatment could identify the possibility of an anterior open bite failure.

### Limitations

The main limitations of this study are its retrospective nature, the small sample size and the difference in gender distribution. As reported in literature [35, 36] when using GMM the power analysis cannot be applied. GMM does not provide any information on size described with numerical values. All the shape variations are averaged, and size information is left out of the Procrustes space. Therefore, there are no numerical ranges available to properly evaluate the sample size. Moreover, in younger children, anterior open bite can be caused by one factor or a combination of factors such as sucking habits, enlarged tonsils or adenoids, tongue position, constricted maxilla, and skeletal open bite growth pattern [37]. The presence of oral habits must always be evaluated before treatment and the elimination of persistent sucking habit must be the first therapeutic goal. In our study this objective was reached by using the QH/C. To the contrary, the skeletal open bite malocclusion needs a different therapeutic approach and in our research these subjects were treated by RME/BB protocol. The presence of different aetiology of AOB and of two different treatment could represent another limitation of the study.

## Conclusion

The increased antegonial notch depth associated with the increased mandibular length and the increased gonial angle could be responsible of relapse of early orthodontic treatment in open bite growing subjects. In conclusion, these skeletal features could help the clinicians to intercept long-term mandibular features of AOB relapse.

## Supplementary Information

**Additional file 1: Supplementary Table 1.** Cephalometric measurements and mean values of the AOB group at T1.

## Data Availability

The datasets used and/or analyzed during the current study are available from the corresponding author on reasonable request.

## Abbreviations

AOB: anterior open bite
OVB: overbite
CS: Cervical Stage
CSV: Cervical Stage Valutation
RG: Relapse Group
SG: Success Group
RME: Rapid Maxillary Expander
BB: Bite Block
QHC: Quad-Helix Crib
T1: pre-treatment phase
T2: post-treatment phase
GMM: Geometric Morphometric analysis
PCA: principal component analysis
