# Supplementary material for: Mandibular morphometric analysis in open bite early treatment relapse subjects: a retrospective observational pilot study
Source: BMC Oral Health. 2022 Dec 1;22:555. doi: 10.1186/s12903-022-02546-y (PMC9714178; doi:10.1186/s12903-022-02546-y)
Supplement: Supplementary file 1 — Additional file 1: Supplementary Table 1. Cephalometric measurements and mean values of the AOB group at T1. [file 12903_2022_2546_MOESM1_ESM.docx]

Supplementary Table 1: Cephalometric measurements and mean values of the AOB group at T1

| **Cephalometric variables** | **Mean** |
| --- | --- |
| SNA (°) | 78.72° |
| SNB (°) | 75.92° |
| ANB (°) | 2.8° |
| Wits appraisal (mm) | 0.27 mm |
| SN ^ PP (°) | 9.90° |
| PP ^ MP (°) | 37.7° |
| ANS – Me (mm) | 60.08 mm |
| NGo^GoMe (°) | 80.64° |
| Co-Gn (mm) | 96.25° |
| Antegonial Notch Depth (mm) | 1.79 mm |
